# Deciphering exterior: building energy efficiency prediction with emerging urban big data

**DOI:** 10.1038/s42949-026-00348-7

**Published:** 2026-02-04

**Authors:** Maoran Sun, Ce Hou, Qiaosi Li, Fan Zhang, Ronita Bardhan, Qunshan Zhao

**Affiliations:** 1 Sustainable Design Group, University of Cambridge, Cambridge, UK; 2 Department of Civil and Environmental Engineering, The Hong Kong University of Science and Technology, Hong Kong, China; 3 Urban Big Data Centre, School of Social and Political Sciences, University of Glasgow, Glasgow, UK; 4 Institute of Remote Sensing and Geographical Information System, School of Earth and Space Sciences, Peking University, Beijing, China

**Keywords:** Engineering, Energy and society, Geography

## Abstract

In the UK, 28 million households consume 25% of the total energy and contribute to 25% of the carbon emissions. It is vital to focus on sustainability and energy efficiency within the building sector for decarbonizing purposes. However, traditional methods such as simulations or on-site inspections are time-consuming and labor-intensive. In this research, we propose a novel methodology framework for estimating building energy efficiency using only external and widely existing data. We have designed and trained an end-to-end multi-channel deep learning model utilizing high-resolution thermal infrared and optical remotely sensed images, street view images, socio-economic indicators, and building morphological data. Validated in Glasgow and Edinburgh, the model achieved F1 scores of 0.64 and 0.69. Further analyses surprisingly suggest that more deprived neighborhoods tend to have better building energy efficiency. The study highlights how widely available data and AI can provide scalable, global solutions for advancing the net-zero agenda.

## Introduction

Monitoring energy consumption in buildings is of great importance for sustainable urban development globally. The building sector accounts for 40% of the total global primary energy consumption, and more than 50% of building energy consumption comes from energy operation^1–3^. Accurate estimation and planning of energy performance in buildings is therefore essential for sustainable urban development. The importance of energy sustainability in cities worldwide is repeatedly emphasized in Goal 7 (Ensure sustainable energy supplies and promote universally accessible, reliable, sustainable, and modern energy services for all) and Goal 11 (Make cities and human settlements inclusive, safe, resilient, and sustainable) of the Sustainable Development Goals of the United Nations. Energy Performance Certificate (EPC) was introduced in 2002 by the Energy Performance of Buildings Directive (Directive 2002/91/EC) as a compulsory requirement for EU member states in the context of constructing, selling, or renting a building or dwelling^4^. As a result, the UK implemented the EPC in 2008 to require buildings to provide an EPC when they are sold or rented to assess the building’s energy efficiency and CO_2_ emissions, and to provide advice to improve the building’s energy efficiency and reduce its environmental impact.

Assessing the energy efficiency of buildings presents a formidable challenge in both real-world and academic contexts. The conventional approach to assessing the energy efficiency of houses predominantly relies on human audit, necessitating the organization of regular visits and measurements by a substantial number of staff to evaluate the energy efficiency of residential properties within a given city^5^. While this method yields a relatively precise assessment, it is both time-consuming and labor-intensive. Although the policy for producing EPC has existed for more than 15 years, there is still around 50% of buildings in the UK that do not have a valid EPC. Other statistical or building simulation methods can be applied to estimate building energy efficiency, but the challenge also arises from the complex interplay between energy consumption and various factors, such as the esthetics of the building, the materials used, the insulation level, and even the age of the structure^5,6^. Consequently, classifying a building’s energy efficiency solely through statistical or simulation-based methods becomes increasingly challenging due to the sheer number of buildings requiring assessment and the time-intensive nature of traditional evaluation processes.

With the increase of computational capability and more available data sources, deep learning methods have become increasingly popular in the field of building energy-related studies in recent years^6–11^. The advantage of this method is that it can combine multi-source and non-traditional data, such as building external and internal images through nonlinear means to perform spatio-temporal dynamic assessments of building energy efficiency. To overcome these challenges and adopt the emerging technological development, we propose a model to evaluate building energy efficiency with only external data from the individual buildings, based on an end-to-end deep learning architecture. This framework integrates only external data of buildings, including aerial high-resolution thermal infrared (TIR) images, street view images (SVIs), aerial photography, and building morphological features. By harnessing the multi-source information, our model extracts pertinent features and subsequently classifies them into High-performance and Low-performance buildings based on the definition from the Energy Performance Certificate. We implement and test the framework in two major cities in Scotland, including Glasgow and Edinburgh, and predict the building energy efficiency for those buildings without human-generated EPCs. The model’s ability to conduct large-scale evaluations automatically significantly enhances overall efficiency in this context.

The overarching goal of our research is as follows: (1) We propose a novel method to facilitate large-scale automated energy efficiency surveys, providing valuable insights into the energy performance of buildings; (2) we propose a scalable framework to estimate and predict building energy performance with only widely available external datasets; (3) we analyze the relationships between building energy efficiency and deprivation level, which helps us better understand the area that needs further building retrofitting in two major Scottish cities. Ultimately, such a knowledge and analysis framework can be applied worldwide and could provide effective recommendations for housing retrofitting policy and construct a more sustainable city.

## Results

### Building energy performance estimation with only external data

To explore the potential of external data in estimating building energy performance, we compiled datasets and trained models for both Glasgow and Edinburgh. The datasets are organized for two cities separately. These datasets contain buildings with all attributes described from the Data Section Data, including energy efficiency rating, SVI, TIR image, aerial image, building morphological attributes, and socioeconomic indicators.

The Glasgow dataset contains 55,018 samples, with 26,859 being high-efficiency buildings and 28,159 being low-efficiency buildings. The Edinburgh dataset includes 6631 high-efficiency buildings, 7037 low-efficiency buildings, and a total size of 13,668 buildings. Each sample corresponds to an individual building and includes processed GSV data, aerial TIR imagery, aerial photography, general building attributes, and the associated energy label from existing EPCs. The dataset is split into train, validation, and test sets with the proportions of 70%, 15%, and 15%.

Two models are trained based on the training split of these two datasets. The model and entire computation are implemented in Python 3.9 and PyTorch on an Ubuntu 18.04 platform. The trained models are then evaluated with the test set held with micro-averaging precision, recall, and F1-Score.

Table 1 presents the confusion matrix for the end-to-end models. The overall F1 scores for the Glasgow and Edinburgh models are 0.64 and 0.69, respectively. Specifically, the precision for High Efficiency houses in Glasgow is 0.81. This implies that if a house is predicted as High Efficiency by our model, there is an 81% chance that it truly belongs to this category. While both models present similar F1 scores, they exhibit different patterns for the two classes. The Glasgow model achieves higher precision for high-efficiency buildings, while the Edinburgh model has better performance for low-efficiency houses.

**Table 1:** Confusion matrix for HtD classification

City	Glasgow		Edinburgh	
	High efficiency	Low efficiency	High efficiency	Low efficiency
High efficiency	**0.81**	0.19	**0.44**	0.56
Low efficiency	0.45	**0.55**	0.19	**0.81**
F1-score	0.64		0.69	

### Minimal data source required for building energy performance estimation

Given the multi-source data and high-dimensional features, it is crucial to understand the contribution of each data source to the prediction. To clarify this, we conducted a model ablation study using various combinations of data sources. The results are displayed in Tables 2 and 3. The first column introduces the combinations of input features, while the subsequent columns present the macro-averaged precision, recall, and F1 score for each model.

**Table 2:** Ablation study of binary model (Glasgow)

Data	Precision	Recall	F1 score
Single source			
SVI	0.71	0.51	0.59
TIR	0.52	0.70	0.60
Aerial	0.67	0.56	0.61
Double sources			
SVI + Aerial	0.73	0.58	0.65
SVI + TIR	0.69	0.62	0.66
Aerial + TIR	0.66	0.56	0.61
Multiple sources			
SVI + Aerial + TIR	0.72	0.57	0.63
SVI + Aerial + TIR + Socio	0.75	0.55	0.64

**Table 3:** Ablation study of binary model (Edinburgh)

Data	Precision	Recall	F1 score
Single source			
SVI	0.60	0.78	0.68
TIR	0.52	0.91	0.67
Aerial	0.59	0.68	0.63
Double sources			
SVI + Aerial	0.63	0.73	0.68
SVI + TIR	0.62	0.67	0.64
Aerial + TIR	0.59	0.71	0.64
Multiple sources			
SVI + Aerial + TIR	0.63	0.70	0.66
SVI + Aerial + TIR + Socio	0.61	0.81	0.69

The ablation study reveals that, in general, incorporating more data sources enhances prediction performance, with two-source and multi-source models delivering similar results. In the single-source group, the SVIs alone achieved the lowest F1 score of 0.59 for Glasgow and the highest of 0.68 for Edinburgh. Both high-resolution TIR images and aerial images individually achieve F1 scores above 0.6. Among the two-source models, all models attain a precision of over 0.6, and most achieve an F1 score exceeding 0.65.

### Building energy performance prediction

To better illustrate the framework and imply the policy intervention, we apply our model to predict energy performance for houses without EPC labels in Glasgow and Edinburgh. Following the same steps described in the Data Section Data, a dataset is organized for both cities. The prediction dataset contains 90,954 and 45,090 buildings for Glasgow and Edinburgh, respectively.

We apply our trained model from two cities over the prediction datasets. The prediction is made on the building level. For both cities, the majority of buildings are predicted to be Low-efficiency, corresponding to EPC labels D to G. Specifically, 50,735 out of 90,954 buildings are predicted as low-efficiency in Glasgow. For Edinburgh, 33,164 out of 45,090 are predicted as Low Efficiency.

While predictions are made for the building instance level, policy intervention and actions are usually made on an administrative unit level. To better inform the policy and understand the pattern across the city, the results are aggregated into data zones (census units in Scotland) level to match with the Scottish Index of Multiple Deprivation (SIMD), the deprivation indicator. Our results are spatially joined to the data zone and aggregated to 4819 and 2488 data zones in Glasgow and Edinburgh. For each data zone, a building energy performance indicator is calculated by dividing the count of high-efficiency buildings to the total buildings in the prediction dataset.

### Co-occurrence of deprivation and energy inefficient houses

**Fig. 1:**
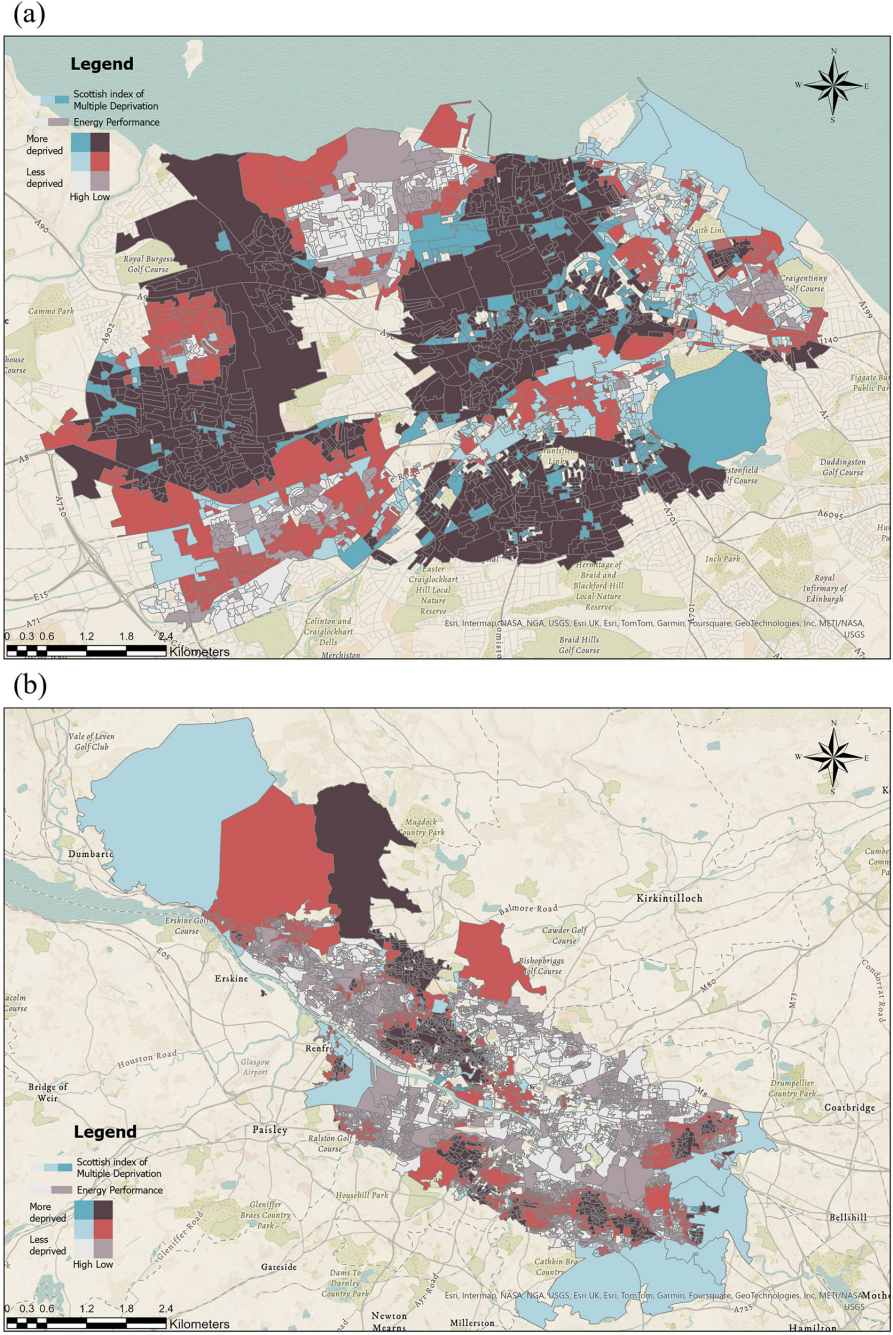
Bivariate factor map about relationships between neighborhoods’deprivation (SIMD) and energy performance. **a** Edinburgh; **b** Glasgow. Areas with high deprivation (SIMD deciles 8–10) and low energy performance (EPC ratings **D**–**G**) are highlighted in dark red, indicating zones of dual disadvantage.

To better understand the relationship between predicted building energy performance and deprivation, a correlation analysis is performed for SIMD and building energy performance indicators. We use the rank of SIMD to represent the deprivation status for each data zone. For the SIMD ranking, 1 represents the most deprived, and a larger number means less deprivation. A Pearson correlation is calculated for both Glasgow and Edinburgh. Both correlations exhibit significant relationships. The correlation coefficients are −0.52 and −0.35 for Glasgow and Edinburgh. It shows that deprivation and High Efficiency building energy are positively correlated, meaning more deprived areas have better building energy performance. Figure 1 illustrates the relationships between neighborhoods’ SIMD and energy performance. In this map, SIMD deciles 1–3 are classified as areas of low deprivation, 4–7 as areas of moderate deprivation, and 8–10 as areas of high deprivation. In terms of energy performance, the primary EPC ratings of buildings within a region serve as the evaluation criterion, with ratings A-C categorized as high energy performance areas and D-G as low energy performance areas. Areas where high deprivation coincides with low energy performance are highlighted in dark red in the visualization, emphasizing the spatial distribution of this dual disadvantage.

**Fig. 2:**
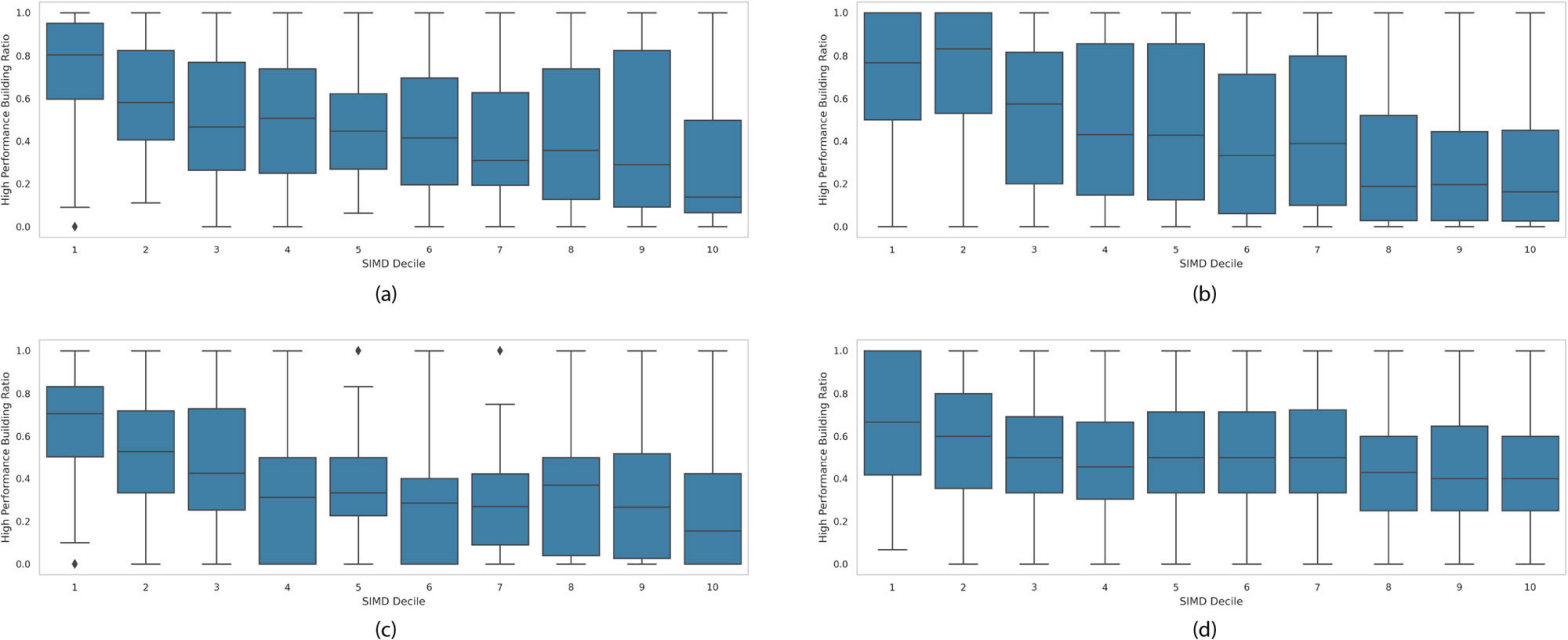
Boxplots of neighborhood-level energy performance and SIMD. Upper Left: Glasgow Prediction; Lower Left: Glasgow Ground Truth; Upper Right: Edinburgh Prediction; Lower Right: Edinburgh Ground Truth. Both cities show similar distributions, with the most deprived neighborhoods exhibiting the highest energy performance.

Much previous literature links socially disadvantaged groups with energy-inefficient houses. However, our results show that the deprivation level is positively correlated with high energy efficiency buildings, at least in our study area. This contradicts our expectations that rich neighborhoods will have better building energy performance. Further analysis is performed to examine the validity of our observation. In Fig. 2, we show both the ground truth and prediction of energy performance at the neighborhood level by SIMD deciles for both cities. It reveals that for both cities, ground truth and prediction results have similar distributions, with most deprived neighborhoods having the highest energy performance.

Recently, Buyuklieva, Boyana, et al.^12^ reached a similar conclusion while studying the variations of the EPC data. They find little evidence to suggest that more affluent groups are preferentially choosing more efficient properties or taking action at higher rates–quite the contrary. This might be because the deprived neighborhoods have been extensively targeted for energy efficiency improvement policies or programs. Also in affluent area, the cost of energy is not as sensitive as the deprived households, and sometimes people live in affluent area wish to maintain their special building architectural styles without putting extra external building insulation.

## Discussion

With buildings being the largest energy consumption sector, improving their efficiency helps reduce greenhouse gas emissions and supports climate change mitigation and carbon-neutral policies. The UK aims to upgrade private rented properties to EPC band C or higher by 2030^13^. Property owners benefit from understanding current ratings and the impact and cost of renovations, while policymakers can identify properties needing improvement for compliance.

Facing the challenge of estimating the whole building stock in the UK, our study uses only external data to classify and predict the building energy performance without detailed inspections of houses. Combining the external data and deep learning, our classification can achieve an F1-score of 0.64 and 0.69 for Glasgow and Edinburgh. Further ablation study discussed how each data source contributes to the prediction. The ablation study demonstrates that, overall, incorporating additional data sources improves prediction performance. Within the single-source category, the SVIs alone achieve the F1 scores of 0.59 for Glasgow and 0.68 for Edinburgh. Both high-resolution TIR images and aerial images individually attain F1 scores above 0.6. For the two-source models, all configurations achieve a precision greater than 0.6, and most exceed an F1 score of 0.65.

By focusing on external data to predict building energy performance, our research harnesses the power of widely available, cost-effective, and comprehensive data sources. This approach not only enhances the scalability and consistency of our predictive models but also ensures adaptability and improved prediction accuracy across various building types and locations. It demonstrates that SVIs, aerial images, and high-resolution TIR images can provide valuable information, not only about visible attributes like building style but also about intrinsic characteristics such as energy efficiency. By combining multi-source image data with building characteristics and socioeconomic factors, the study achieves relatively high performance in estimating property-level energy efficiency.

There are limitations in this study that could be addressed in future research. One of the limitations is the data quality of the EPC dataset. Our work heavily depends on the EPC dataset, and it is well-documented that there are uncertainties in the existing EPC dataset, particularly regarding the gap between estimated and actual energy performance^14–17^. However, with the increasing coverage of EPC, European countries have established standards for quality assurance. Future work can focus on gradually minimizing these uncertainties in the dataset by comparing the EPC records with energy use intensity and energy consumption. We anticipate more related data will become available in the future, such as the data combining EPC, smart meter data, indoor environment, and social survey from the Understanding Society Innovation Panel (https://www.understandingsociety.ac.uk/participants/projects/smartmeter/) (https://www.understandingsociety.ac.uk/participants/projects/sensor/). With more and more linked building indoor and outdoor data available, better modeling and validation can be done in the future.

Another limitation is the limited availability of high-resolution TIR images, which restricts the generalizability and transferability of our method to other contexts. Currently, the scarcity of this data poses a challenge for broader applications. However, the increasing number of high-resolution satellites with TIR sensors being launched is expected to enhance data availability in the near future. As more high-resolution TIR images become accessible, we anticipate that more future work in other areas of the world will verify the applicability and robustness of our method, enabling its use in a wider range of contexts and locations. In addition to the limited availability of TIR images, we acknowledge the potential bias and ambiguity in interpreting the thermal signals. While higher surface temperatures generally indicate heat loss and thus poor insulation, lower surface temperatures do not necessarily imply better energy performance. They may instead reflect other factors such as limited indoor activity, inadequate heating, or low occupancy. To mitigate this issue, we conducted the flight campaign in Glasgow during the winter season, when buildings are typically in full heating operation. This timing was intended to enhance the visibility of heat loss patterns in the thermal imagery. For Edinburgh TIR data, we unfortunately collected the data at night during the summer season rather than the optimal winter window, which might introduce potential data issues. We recognize that external thermal data alone may not fully capture the complexity of internal building performance.

## Methods

Our study proposes an end-to-end multi-channel deep learning framework to fuse the Aerial TIR images, SVIs, aerial images, and building characteristics extracted from building morphology to estimate the building energy efficiency. Model evaluation methods and model interpretation are proposed to evaluate the effectiveness of our framework. An ablation test is conducted to understand the contributions from different data sources. We used the existing EPC data as our validation datasets. Finally, the model is applied to buildings with no ground truth EPC label to understand building energy performance in both cities at a large scale.

### Data

In this section, we describe the datasets used in our study. The primary sources include Energy Performance Certificates (EPC), aerial TIR imagery, SVI, aerial photographs, and socioeconomic data.

As one of the most fundamental data sets within this study, the building footprints are used as the reference for linking different data sources. The building footprint data is obtained from the UK Buildings dataset (https://digimap.edina.ac.uk/). The dataset provides 2D boundaries of building footprints across the whole UK.

The EPC data captures precise energy performance details of the properties, including the dimensions and configuration of the property, and the approaches for its construction, insulation, heating, ventilation, and lighting. With this information, the EPC assessors apply a calculation technique to project the properties’ energy consumption to an “Energy Efficiency Rating”. The rating ranges from A to G, with A representing the highest efficiency and G representing the lowest efficiency. In light of the UK government’s goal to upgrade private rented properties to an EPC band of C or higher by 2030, we classified EPC ratings into “High Efficiency” (original bands A–C) and “Low Efficiency” (original bands D–G).

UK government provides the open-access EPC data^3,18^. The EPC data for Glasgow and Edinburgh is available through the Scottish Government’s data portal (https://statistics.gov.scot/data/domestic-energy-performance-certificates). In this study, we incorporated the EPC records from October 2012 to September 2023. We used the zip codes of Glasgow and Edinburgh to extract data from the whole Scottish EPC database. The addresses of properties from EPC data were then geocoded to obtain the accurate latitude and longitude with Google Geocoding API (https://developers.google.com/maps/documentation/geocoding/overview). The geocoded properties with EPC information were then spatially joined with building footprints to perform building-level prediction. For buildings with more than one property and various EPC ratings, the most frequently occurring rating (mode) is adopted as the grade for the building.

Aerial TIR images were acquired from airplanes at nighttime using a broadband and push-frame TIR imager (MICROTABI640) with a spectral range of 3.7–4.8 µm. Two flight campaigns were conducted within Glasgow city between 20:01-21:13, 7th March 2023, and between 21:14-22:04, and 20th April 2023, in GMT+0 respectively. Considering the data quality and coverage, the data from 7th March was selected for further analysis. Meanwhile, two ground sensors were deployed at a height of 1.5 m above ground and one ground sensor was placed on a residential building rooftop within the flight area to measure temperature and relative humidity during the flight mission. During the March Glasgow flight campaign, the air temperature is −1 °C. A flight campaign was carried on within Edinburgh city from 23:34, 26th to 02:05, 27th July 2022 in GMT+1. TIR images were captured at approximately 2438.4 m above ground level with 50% of image overlap. For the Edinburgh flight campaign, the air temperature is +11 °C. Image preprocessing includes georectified, orthorectified, radiometric, bad pixel corrected, and emissivity-adjusted calibration. All of the preprocessing was conducted by the vendor. As a result, TIR images of relative emissivity that can indicate the energy radiated from the ground surface in 3.5 m spatial resolution were obtained for this study.

SVIs serve as a street-level digital reflection of constructed environments, offering a crucial tool for the exploration and analysis of architecture and urban areas. SVIs are proven to have the ability to accurately describe the buildings in the city. In this study, we collect SVIs based on the orientation of the façade of the building to represent the exterior characteristics of the buildings^19^.

SVIs are collected through the Google Street Map platform^20^. Google provides high-quality panoramic SVIs that cover almost every driving street in our study area. As one of the most popular SVI sources in the world, Google SVIs support a series of urban research from the greenery audit to the urban socio-economic atmosphere^21–25^. We request Google SVIs for all individual buildings for further analysis. The detailed process of the request is illustrated in ref. ^19^.

In this research, aerial images are employed to capture the surrounding context of the building structures. The data is downloaded from Getmapping Plc, Digimap (https://digimap.edina.ac.uk/). The aerial imagery is captured at a resolution of 25 cm. We downloaded the aerial image tiles that overlap within our study area. Then the tiles are cropped according to the bounding box of each building.

Two sources of socioeconomic data are involved in this research. The first is census data and used as input to the deep learning model. Socioeconomic indicators are introduced as a proxy for the human factors of buildings’ energy usage behavior. All socioeconomic data is downloaded through EDINA Society Digimap Service (https://digimap.edina.ac.uk/). The data contains Social grade, Highest level of qualification, Population density, Occupancy rating, Long-term health problem or disability, Household composition, Economic activity, Country of birth, Families with dependent children, Car or van availability to household, Age by single-year and Accommodation type. The census attributes are from the 2011 census. The socioeconomic information gathered is specific to the Output Area level. Each building is allocated to its corresponding Output Area, and the socioeconomic characteristics are then linked to these buildings.

The second source of socioeconomic data is the SIMD. It is used in this paper to correlate with our prediction results. SIMD is a relative measurement of deprivation in Scotland. If a region is labeled as ’deprived,’ it may indicate not only low income among residents but also a scarcity of resources or opportunities. The SIMD assesses the level of deprivation within a locality across seven domains: income, employment, education, health, access to services, crime, and housing. SIMD data is obtained from the Scottish Government website (https://www.gov.scot/collections/scottish-index-of-multiple-deprivation-2020/). In this study, the ranking of deprivation for each data zone is used to represent the deprivation level, with one being the most deprived and larger numbers related to less deprivation.

### End-to-end deep learning model design

**Fig. 3:**
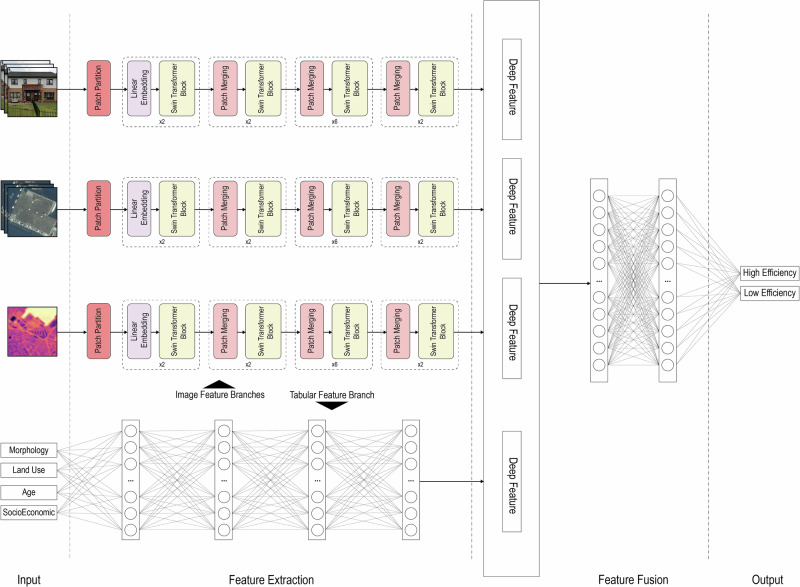
Architecture of the proposed deep learning model for energy efficiency classification. The network integrates three image streams (TIR, street view, and aerial) processed with a Swin Transformer backbone and one descriptive feature stream. Extracted features are fused into a joint vector and passed through fully connected layers to predict energy efficiency categories.

Leveraging the detailed building and image datasets, we develop a deep convolutional neural network tailored for energy efficiency classification. This model is designed to simultaneously extract and learn from the visual cues of TIR images, façade images from Google SVIs, aerial images as well as the general attributes of houses, such as morphological, structural and socioeconomic data of properties. The architecture of our network, as shown in Fig. 3, unfolds in four primary phases: input, feature extraction, feature fusion, and output. It operates through four concurrent streams during the input and feature extraction phases: three for images and another for descriptive features. The image streams are identical and we process image data using a Swin Transformer^26^ framework as a backbone to extract a 1024-dimensional feature vector for subsequent fusion. Meanwhile, the descriptive feature stream employs a fully connected network with four hidden layers, generating a 256-dimensional feature vector. In the feature fusion phase, these stream vectors are merged into a single 3328-dimensional feature vector. This comprehensive feature vector is then fed into a fully connected network with two hidden layers, culminating in classifying the building’s energy efficiency into specific categories.

We employ distinct initialization strategies for the image and tabular feature branches. The image branches are initialized with weights pre-trained on the ImageNet dataset^27^, a widely used dataset for recognizing common objects such as vehicles, buildings, and street signs. These pre-trained weights enable the model to understand and extract information about objects and scenes within the images, facilitating faster network convergence and reducing training time. In contrast, the tabular feature branch is initialized randomly due to its small number of parameters and simple structure.

A customized loss function is employed in the end-to-end deep learning model. Rather than calculating the loss solely for the final output, a negative log-likelihood (NLL) loss is computed for each branch of the model. The losses from all individual branches, along with the final output, are then aggregated. This approach ensures that each branch contributes effectively to the overall prediction.

### Model evaluation and ablation test

The trained model is then evaluated with the test set. The evaluation metrics adopted in our study are micro-averaged precision, recall, and F1 score. Micro averaging considers each observation as weighted equally rather than each class. The entire dataset is treated as a single aggregate result, computing one overall metric instead of averaging multiple class-specific metrics. Recall is determined by dividing the number of true positives (TP) by the sum of TPs and false negatives (FN). Precision is calculated by dividing the number of TPs by the sum of TPs and false positives. The F1-score represents the harmonic mean of precision and recall.

Instead of only focusing on a comprehensive model, we also decipher the model to understand how different data sources contribute to the prediction. Combinations of different data sources are used to train multiple models. Three groups of single-source, double-source, and multiple-source models are trained and evaluated. To ensure consistent results and minimize randomness during experiments, we maintain the same split of training, validation, and test datasets of 70%, 15%, and 15% across all models.

### Model interpretation and prediction

Currently, EPC covers about 50% of UK domestic buildings^7^. To validate the effectiveness of our framework and apply the classification model, we create a dataset by combining all the buildings covered by our high-resolution TIR images, SVIs, and aerial images, but missing EPC labels for both Glasgow and Edinburgh. The end-to-end classification model performs predictions to all the buildings in the dataset and classify them into buildings with High Efficiency and Low Efficiency.

## Data Availability

The EPC, Building footprint, aerial image, and socioeconomic data are acquired under an educational license. EPC data is available at https://www.scottishepcregister.org.uk/. The Building Footprint, aerial image, and socioeconomic data are available at https://digimap.edina.ac.uk/. The aerial thermal image was obtained from SatVu’s aerial survey programme, and the images can only be shared with prior written consent from SatVU.

## Code availability

The codes can be accessed at https://github.com/MaoranSun/Deciphering-Exterior.
